# Assessing the practicality of using a single knowledge‐based planning model for multiple linac vendors

**DOI:** 10.1002/acm2.13704

**Published:** 2022-07-05

**Authors:** Raphael J. Douglas, Adenike Olanrewaju, Lifei Zhang, Beth M. Beadle, Laurence E. Court

**Affiliations:** ^1^ Department of Radiation Physics The University of Texas MD Anderson Cancer Center Houston Texas USA; ^2^ Department of Radiation Oncology Stanford University Palo Alto California USA

**Keywords:** automated planning, knowledge‐based planning

## Abstract

**Purpose:**

Knowledge‐based planning (KBP) has been shown to be an effective tool in quality control for intensity‐modulated radiation therapy treatment planning and generating high‐quality plans. Previous studies have evaluated its ability to create consistent plans across institutions and between planners within the same institution as well as its use as teaching tool for inexperienced planners. This study evaluates whether planning quality is consistent when using a KBP model to plan across different treatment machines.

**Materials and methods:**

This study used a RapidPlan model (Varian Medical Systems) provided by the vendor, to which we added additional planning objectives, maximum dose limits, and planning structures, such that a clinically acceptable plan is achieved in a single optimization. This model was used to generate and optimize volumetric‐modulated arc therapy plans for a cohort of 50 patients treated for head‐neck cancer. Plans were generated using the following treatment machines: Varian 2100, Elekta Versa HD, and Varian Halcyon. A noninferiority testing methodology was used to evaluate the hypothesis that normal and target metrics in our autoplans were no worse than a set of clinically‐acceptable baseline plans by a margin of 1.8 Gy or 3% dose‐volume. The quality of these plans were also compared through the use of common clinical dose‐volume histogram criteria.

**Results:**

The Versa HD met our noninferiority criteria for 23 of 34 normal and target metrics; while the Halcyon and Varian 2100 machines met our criteria for 24 of 34 and 26 of 34 metrics, respectively. The experimental plans tended to have less volume coverage for prescription dose planning target volume and larger hotspot volumes. However, comparable plans were generated across different treatment machines.

**Conclusions:**

These results support the use of a head‐neck RapidPlan models in centralized planning workflows that support clinics with different linac models/vendors, although some fine‐tuning for targets may be necessary.

## 1 INTRODUCTION

Intensity‐modulated radiation therapy treatment planning is a challenging and time‐consuming process that can vary in quality between planners across institutions^1^, ^2^ and even within the same institution.^3^ Knowledge‐based planning (KBP) has been shown to be effective in creating high‐quality treatment plans,^4^, ^5^, ^6^, ^7^, ^8^, ^9^ reducing variability between planners,^9^ and evaluating plan consistency and quality.^2^, ^3^, ^11^, ^12^, ^13^, ^14^ These factors make KBP models ideal for centralized planning systems, such as the radiation planning assistant (RPA).

The RPA is a web‐based automated treatment planning system that is being developed to provide high‐quality contours and treatment planning to clinics with limited resources around the world. The RPA's development has been described in previous works.^5^, ^15^, ^16^, ^17^, ^18^, ^19^, ^20^, ^21^, ^22^ Through the Eclipse application programming interface, the RPA uses RapidPlan (Varian Medical Systems, Palo Alto, CA), a commercial KBP system, to generate and optimize treatment plans for various cancer sites.

Since we aim to provide plans generated by the RPA to multiple clinics around the world, we needed to evaluate how plan quality is affected when a single RapidPlan model is used to generate plans with different treatment machines that have different beam qualities and multileaf collimator (MLC) characteristics. In this study, we started with a KBP approach that was developed for head and neck treatment on a Varian 2100 series linac, with plan quality validated by radiation oncologists from multiple institutions. We then used the same approach to create volumetric‐modulated arc therapy (VMAT) plans for treatment on Versa HD (Elekta) and Halcyon (Varian) treatment devices. The plans were then dosimetrically evaluated for quality by comparing them to our baseline, physician‐reviewed autoplans. Previous studies have shown the robustness of KBP models to tumor location, treatment modalities, and institutional protocols,^23^, ^24^ but to our knowledge, this is the first study to evaluate a RapidPlan model across different treatment machines. This comparison is important for centers (or treatment planning services) where there is a need to accommodate different models and vendors.

## 2 METHODS

### 2.1 Patient data

For this analysis, a cohort of 50 patients with head and neck cancer was retrospectively collected and de‐identified. This study was approved by the institutional review board. All patients were previously treated using VMAT. The patients’ original, physician‐drawn targets, and normal tissue contours, along with the original CT scan and dose prescription, were used in autoplan generation. The patients had primary tumors from various subsites such as the nasopharynx, oropharynx, and oral cavity (Table 1).

**TABLE 1:** Breakdown of the patient cohort by site and high‐dose planning target volume (PTV1) range

Primary site	Number of patients	Range of PTV1 dose, Gy
Oropharynx	21	60–70
Oral cavity	8	60–70
Larynx	3	70
Hypopharynx	8	60‐68
Nasopharynx	10	60‐70

### 2.2 Plan optimization

The RPA uses a RapidPlan model to optimize HN plans in Eclipse Treatment Planning System (Varian Medical Systems, Palo Alto, CA). This model was developed using the vendor‐provided Washington University HN RapidPlan model as a starting point. Additional planning objectives, maximum dose limits, and additional planning structures were added to the model. The model was refined through iterative testing and physician feedback using a set of validation patients that were not included in this study. The full planning strategy is outlined in a previous publication by our group.^5^ The model was optimized for a Varian 2100 linac.

### 2.3 Baseline plans

In a previous study by our group, a set of Varian 2100 plans was generated for our patient cohort using Eclipse version 15.5—in which the autoplans were compared to the original, clinically‐approved plan in a blinded review by physicians—and scored for clinical acceptability based on contour quality and dose coverage.^5^ From this study, 49/50 of the autoplans were deemed clinically acceptable by the reviewers. These physician‐reviewed autoplans were dubbed our baseline plans and were used as a basis for our comparison.

### 2.4 Plan generation

Since the creation of our baseline plans, we upgraded Eclipse to version 15.6. Three plans were generated per patient in our cohort using the following machines: Varian 2100, Elekta Versa HD, and Varian Halcyon. We decided to re‐plan the Varian 2100 cases in Eclipse v15.6 in order to verify that any dose differences seen were not the result of the difference in Eclipse versions. The plans generated in Eclipse v15.6 were dubbed our experimental plans.

The Varian 2100 (both baseline and experimental) and Versa HD plans consisted of three 360° coplanar treatment arcs with collimator angles of 15°, 345°, and 90°. The Halcyon plans consisted of four treatment arcs with collimator angles of 0°, 45°, 90°, and 315°. Three of the 50 patients had two planning target volume (PTV) dose levels, while the other 47 had three PTV dose levels.

### 2.5 Evaluation process

To determine the quality of our experimental plans, we used a one‐sided Mann‐Whitney *U* test at 95% confidence to determine whether the experimental doses were noninferior to the baseline doses by a margin, *δ*, we determined based on clinical judgement.^25^, ^26^, ^27^ For a given dose‐volume histogram (DVH) metric, let *M*_B_ be the median value for the baseline plans, and *M*_E_ be the median value for the experimental plans. For DVH metrics in which lower values are better, we have the following hypotheses:

$$\begin{array}{c} H_{0}:M_{E}\geq M_{B}+\delta \\ H_{a}:M_{E}<M_{B}+\delta \end{array}$$

For metrics in which higher values are better, we have the following hypothesis:

$$\begin{array}{c} H_{0}:M_{E}\leq M_{B}+\delta \\ H_{a}:M_{E}>M_{B}+\delta \end{array}$$

Experimental plans are considered noninferior if the null hypothesis is rejected (i.e., *p* < 0.05). A 95% confidence interval for *M*_E_‐*M*_B_ was calculated in order to conclude noninferiority. For DVH metrics in which lower values were better, the upper limit of the 95% confidence interval needed to be less than the margin in order to conclude noninferiority. For metrics in which higher values were better, the lower limit of the confidence interval needed to be greater than the margin. For normal structures, D95%, and D98%, *δ* = 1.8 Gy (i.e., 3% of the lowest dose prescription in our dataset); for V95%, V100%, V105%, and V110%, *δ* = 3%. The number of plans that met established clinically accepted dosimetric criteria outlined in Radiation Therapy Oncology Group protocol 1016^28^ was also calculated and compared in order to assess the clinical acceptability of the autoplans.

## 3 RESULTS

### 3.1 Noninferiority

**FIGURE 1:**
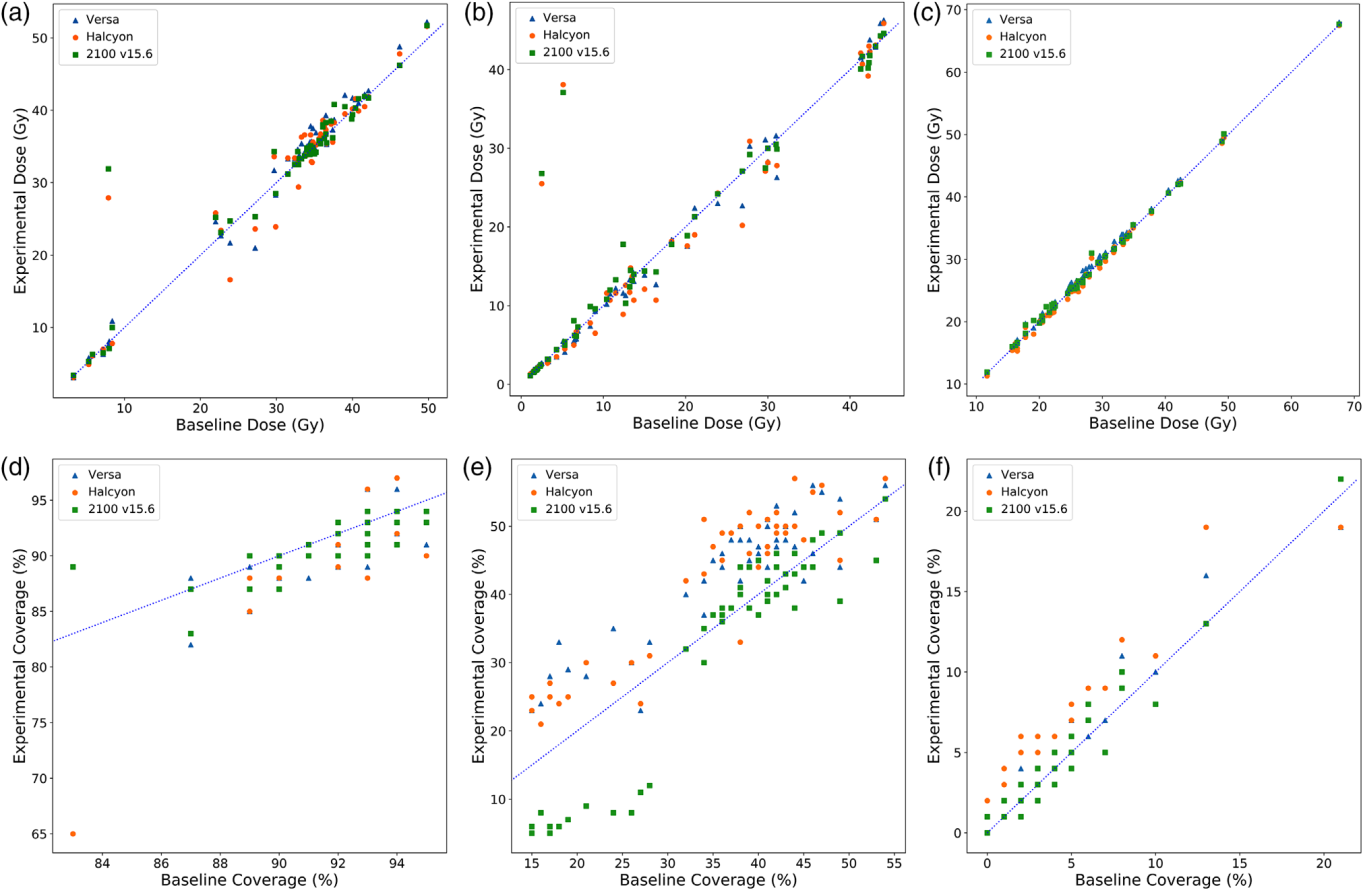
Scatterplots compare the distribution of planned dose to the normal structures for (from top left) the brainstem (a), right cochlea (b), ipsilateral parotid (c), and low‐dose PTV target coverage at 100% (d), 105% (e), and 110% (f) of the prescription dose. The y‐axis represents the planned dose in the experimental plan; the x‐axis represents the planned dose in the baseline plan. The blue triangles represent the Versa planned dose/coverage for a given patient; orange dots represent the Halcyon planned dose/coverage; green squares represent the Varian 2100 (v15.6) planned dose/coverage. Points that lie on the blue line represent patients in which both the experimental and baseline plans gave the same value

**FIGURE 2:**
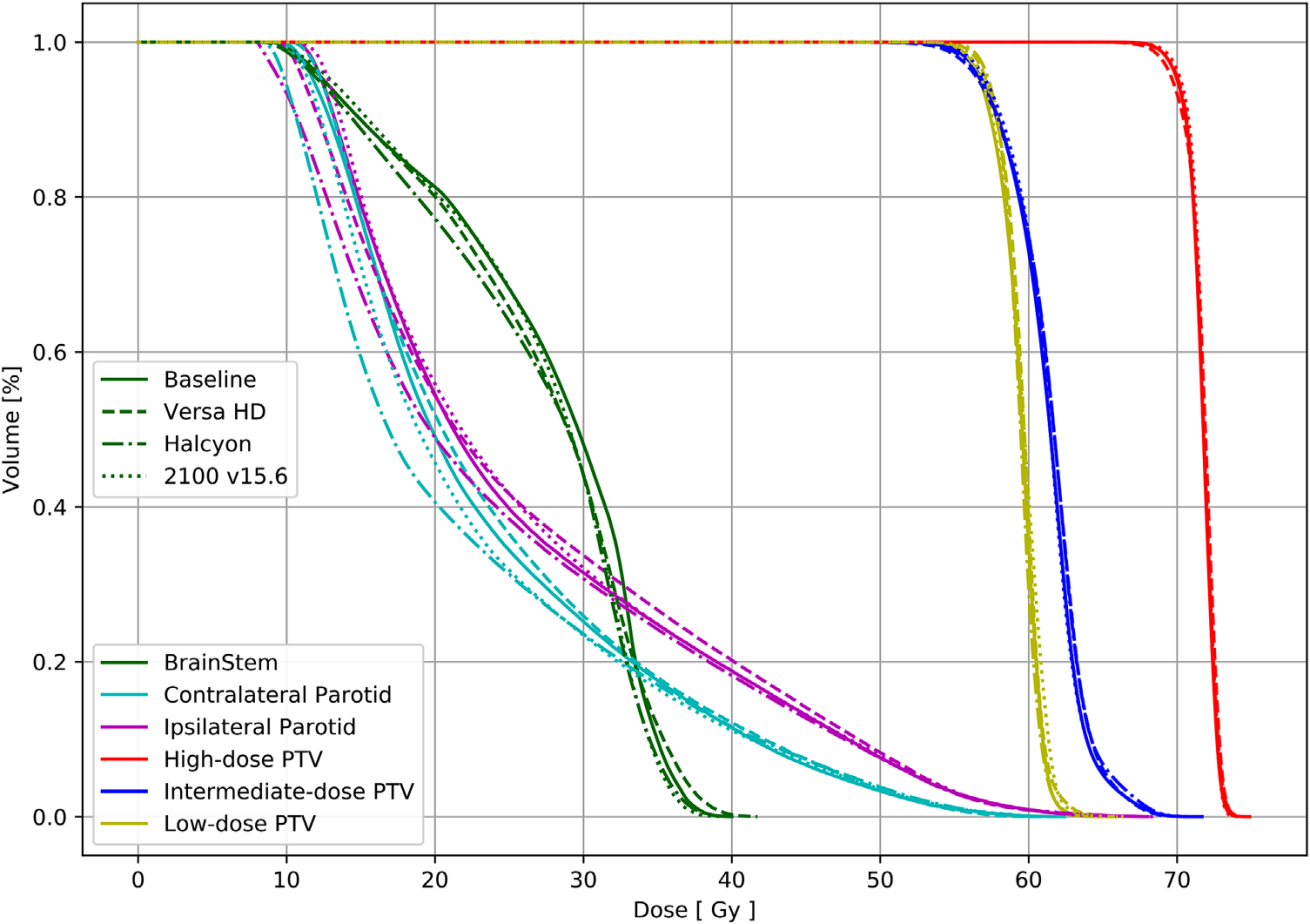
Example dose‐volume histogram (DVH) for one of the patients comparing brainstem, parotids, and target volume

Table 2 shows the confidence interval and *p*‐values from our statistical test for each DVH criteria. The Versa HD was noninferior (*p* < 0.05) for 23 of 34 DVH metrics evaluated; Halcyon was noninferior for 24 of 34 metrics; the Varian 2100 re‐plan was noninferior for 26 of 34 metrics. We were not able to conclude noninferiority for brain, brainstem, both cochleae, ipsilateral parotid, brainstem with 5 mm margins, and spinal cord with 5‐mm margins. For some DVH metrics, we were able to conclude noninferiority for some experimental machines (Versa, Halcyon, 2100 v15.6), but not all. This can be seen in intermediate‐dose V100%, V105%, and low‐dose V105% PTVs, where the *p*‐values for Versa and Halcyon were above 0.05, but for 2100 v15.6, it was less than 0.05. Figure 1 shows the distribution of planned dose to the normal structures for the brainstem, right cochlea, ipsilateral parotid, and low‐dose PTV target coverage at 100%, 105%, and 110% of the prescription dose. The distributions for the brainstem, right cochlea, ipsilateral parotid, and low‐dose PTV target coverage at 105% and 110% show that there was very good agreement between the experimental and baseline plans, with *r*‐squared values greater than 0.82 for all experimental machines. However, there was not very good agreement (*R* ^2 ^< 0.50) for low‐dose target coverage at 100%, due to an outlier. Figure 2 shows a DVH for one of the patients, comparing brainstem, both parotids, and PTVs for the baseline and experimental autoplans. All four plans showed good agreement for those structures.

**TABLE 2:** 95% confidence interval (CI) and *p*‐value of the one‐sided Mann‐Whitney test

Noninferiority test						
	Versa		Halcyon		2100 v15.6	
Structure, metric	95% CI	*p*‐Value	95% CI	*p*‐Value	95% CI	*p*‐Value
Brain, max Dose	(−3.9, 5.0)	0.299	(−2.3, 6.7)	0.516	(−2.9, 6.5)	0.421
BrainStem, max Dose	(−0.7, 2.6)	0.195	(−1.3, 2.2)	0.100	(−1.3, 2.0)	0.072
Chiasm, max Dose	(−0.1, 0.7)	<0.001	(−0.1, 0.6)	<0.001	(−0.3, 0.5)	<0.001
Cochlea_L, max dose	(−3.7, 2.9)	0.123	(−3.3, 3.5)	0.175	(−3.0, 4.3)	0.267
Cochlea_R, max dose	(−3.4, 2.6)	0.099	(−2.9, 3.8)	0.161	(−2.2, 4.8)	0.272
Con_Parotid, mean dose	(−1.3, 2.3)	0.093	(−2.3, 1.3)	0.020	(−1.6, 2.0)	0.064
Eye_L, max dose	(−0.2, 0.6)	<0.001	(−0.4, 0.4)	<0.001	(−0.4, 0.5)	<0.001
Eye_R, max dose	(−0.2, 0.6)	<0.001	(−0.4, 0.5)	<0.001	(−0.4, 0.6)	<0.001
Ips_Parotid, mean dose	(−2.2, 3.4)	0.202	(−3.1, 2.5)	0.099	(−2.5, 3.0)	0.147
Lens_L, max dose	(0.0, 0.6)	<0.001	(−0.1, 0.4)	<0.001	(−0.2, 0.3)	<0.001
Lens_R, max dose	(0.1, 0.6)	<0.001	(−0.1, 0.3)	<0.001	(−0.2, 0.3)	<0.001
Mandible, max dose	(−1.7, 1.3)	0.023	(−1.6, 1.2)	0.025	(−1.5, 1.5)	0.034
OpticNrv_L, max dose	(−0.2, 0.7)	<0.001	(−0.2, 0.6)	<0.001	(−0.4, 0.5)	<0.001
OpticNrv_R, max dose	(−0.2, 0.7)	<0.001	(−0.2, 0.6)	<0.001	(−0.3, 0.5)	<0.001
SpinalCord, max dose	(0.0, 1.5)	0.014	(−0.9, 0.7)	<0.001	(−0.6, 0.8)	<0.001
zBrainStem_05, max dose^a^	(−1.0, 3.1)	0.303	(−0.5, 3.8)	0.477	(−1.0, 3.2)	0.349
zPTV1, D95%	(−1.0, 0.2)	0.003	(−0.8, 0.3)	0.002	(−0.5, 0.5)	0.001
zPTV1, D98%	(−1.3, 0.2)	0.011	(−1.1, 0.3)	0.005	(−0.7, 0.6)	0.002
zPTV1, V100%	(−3.0, ‐1.0)	0.024	(−3.0, 0.0)	0.021	(−1.0, 1.0)	<0.001
zPTV1, V105%	(1.0, 1.0)	<0.001	(1.0, 1.0)	<0.001	(0.0, 0.0)	<0.001
zPTV1, V95%	(0.0, 0.0)	<0.001	(0.0, 0.0)	<0.001	(0.0, 0.0)	<0.001
zPTV2, D95%	(−1.2, 0.3)	0.006	(−1.3, 0.3)	0.007	(−0.7, 0.9)	0.001
zPTV2, D98%	(−1.3, 0.3)	0.008	(−1.4, 0.2)	0.009	(−0.7, 0.8)	<0.001
zPTV2, V100%	(−4.0, ‐1.0)	0.312	(−5.0, −1.0)	0.440	(−1.0, 2.0)	<0.001
zPTV2, V105%	(2.0, 8.0)	0.864	(1.0, 8.0)	0.813	(−5.0, 2.0)	0.030
zPTV2, V110%	(0.0, 1.0)	0.002	(0.0, 2.0)	0.003	(−1.0, 1.0)	<0.001
zPTV2, V95%	(−1.0, 0.0)	<0.001	(−1.0, 0.0)	<0.001	(0.0, 0.0)	<0.001
zPTV3, D95%	(−0.4, ‐0.1)	<0.001	(−0.5, −0.2)	<0.001	(−0.2, 0.0)	<0.001
zPTV3, D98%	(−0.5, ‐0.2)	<0.001	(−0.6, −0.3)	<0.001	(−0.2, 0.1)	<0.001
zPTV3, V100%	(−2.0, ‐1.0)	<0.001	(−2.0, −1.0)	0.002	(−1.0, 0.0)	<0.001
zPTV3, V105%	(5.0, 8.0)	0.978	(7.0, 9.0)	0.997	(−2.0, 0.0)	0.018
zPTV3, V110%	(0.0, 1.0)	<0.001	(1.0, 2.0)	0.018	(0.0, 0.0)	<0.001
zPTV3, V95%	(0.0, 0.0)	<0.001	(−1.0, 0.0)	<0.001	(0.0, 0.0)	<0.001
zSpinalCord_05, max dose^a^	(−0.2, 1.7)	0.133	(−0.9, 1.2)	0.054	(−0.4, 1.5)	0.099

Abbreviations: DX%, doses at the given percent of target coverage; *D*_max_, maximum dose; *D*_mean_, mean dose; VX%, percent of the target receiving the prescribed dose.

^a^ Structure with a 5 mm margin.

### 3.2 Dosimetric criteria

The number of autoplans that met established clinical dosimetric criteria are listed in Table 3, along with the original clinical plans for comparison. All autoplans met clinical recommendations for the spinal cord, brainstem, optic chiasm, optic nerves, PTV1 (V1cc < 117% and V95% > 95%), PTV2 (V95% > 80%), and PTV3 (V95% > 80%).

**TABLE 3:** Number of plans that met clinical dose criteria based on RTOG 1016 for the original clinical plans and the autoplans evaluated in this study

Number of clinical/autoplans that met clinical dose recommendations						
Structure	Constraint	Clinic	Baseline	Versa HD	Halcyon	2100 v15.6
Spinal cord	*Dmax < 45 Gy*	50	50	50	50	50
Brainstem	*Dmax < 54 Gy*	48	50	50	50	50
Ipisilateral parotid	*Dmean < 26 Gy*	29	24	21	25	24
	*V30Gy < 50%*	40	42	40	41	42
Contralateral parotid	*Dmean < 26 Gy*	44	41	41	40	40
	*V30Gy < 50%*	48	47	46	46	46
Cochleae	*Dmax < 35 Gy*	45	40	40	39	40
Optic chiasm	*Dmax < 54 Gy*	49	50	50	50	50
Optic nerves	*Dmax < 54 Gy*	49	50	50	50	50
Lenses	*Dmax < 7 Gy*	44	45	46	47	45
Eyes	*Dmax < 35 Gy*	45	45	45	45	45
Brain	*Dmax < 54 Gy*	34	36	37	37	36
High‐dose PTV	*V1cc < 110%*	48	49	50	48	50
	*V1cc < 117%*	50	50	50	50	50
	*V95% > 95%*	50	50	50	50	50
Intermediate‐dose PTV	*V95% > 100%*	34	16	9	10	13
	*V95% > 80%*	50	50	50	50	50
Low‐dose PTV*	*V95% > 100%*	30	30	15	15	21
	*V95% > 80%*	47	47	47	47	47

Abbreviations: *D*_max_, maximum dose; *D*_mean_, mean dose; PTV, planning target volume; RTOG, Radiation Therapy Oncology Group.

^*^ Three patients were treated with only two PTVs.

## 4 DISCUSSION

The goal of this study was to evaluate the ability of a single RapidPlan model to generate comparable plans across different treatment machines that have different beam and MLC characteristics. Using a RapidPlan model that was optimized for a Varian 2100 linac, we were able to generate plans that were noninferior to our baseline in at least 68% of the DVH metrics evaluated for each set of experimental plans. For the metrics in which we were not able to conclude noninferiority, we conducted an investigation into the reason why the noninferiority criteria was not met. We noticed that there were outliers for the Halcyon and Varian 2100 (v.15.6) machines in the following normal structures: brain, brainstem, right cochlea, left cochlea, and brainstem (with 5‐mm margins). These outliers came from the same two patients, one of them being the patient in which the clinically unacceptable baseline plan was generated for in the aforementioned previous study.^5^ Both of these patients were planned with two PTVs. The Halcyon and Varian 2100 plans for these patients had large dose gradients around the cochleae, and one had a target close to the brain, which led to a large dose gradient around the brain/brainstem. We also noticed that, for these two patients, the Halcyon and Varian 2100 (v.15.6) plans were more comparable to the original plans used treat in the clinic than to our baseline autoplans. The experimental Varian 2100 plans that were generated in Eclipse v15.6 were noninferior in the most DVH criteria. Overall, these plans were noninferior for all 17 DVH criteria for target structures.

The target volumes in our study were less homogeneous for the Versa HD and Halcyon plans than for the baseline plans. In general, these experimental plans had reduced coverage of the prescription dose (V95% and V100%) and larger hotspots (V105% and V110%) at all dose levels. This indicates that some additional fine‐tuning of the plans may be needed when applying the standard RapidPlan approach to different machines with different beam qualities and MLC characteristics. This sort of fine‐tuning, however, has been shown to require minimal effort.^29^

This study has some limitations. The sample size for this study is a limiting factor of our test. This is apparent when looking at the mean dose to the parotids. The treatment machines that we were not able to confirm noninferiority for the parotids each had only one plan with a dose above the 1.8 Gy margin. Additionally, the parotid dose in these plans were less than 1 Gy above the margin. Prior to reproducing this study, we would need to increase the power of our tests by increasing our sample size. While the baseline plans were determined to be clinically acceptable, we are not able to conclude that the same for the experimental plans generated for this study. Furthermore, an inherent weakness with this study is that we can only confirm noninferiority for the model under investigation. However, we can confirm that it is possible to create noninferior plans with a KBP model across different treatment machines. We have also established a methodology for evaluating other KBP models across different treatment machines.

Evaluating the effects of applying a single RapidPlan model to linacs developed by various vendors is important as more automation tools, such as the RPA, are being developed that will allow for centralized planning. It will be important to understand the strengths and weakness of these models as they will be used on multiple linac models and vendors around the world. We have shown in this study there is very good agreement between plans generated with different linac types, although some fine‐tuning of models may be needed to improve target coverage and minimize hotspots.

## CONFLICT OF INTEREST

The authors declare that there is no conflict of interest that could be perceived as prejudicing the impartiality of the research reported.

## AUTHOR CONTRIBUTIONS

Laurence Court, Adenike Olanrewaju, and Lifei Zhang conceived and designed this experiment. Beth Beadle provided clinical guidance for the model used in the experiment. Raphael Douglas carried out the experiment, data collection, and analysis of the data. Laurence Court supervised the findings in this work. Raphael Douglas wrote the manuscript with the support of Laurence Court.
